# Formulation and Optimization of Clotrimazole-Loaded Proniosomal Gel Using 3^2^ Factorial Design

**DOI:** 10.3797/scipharm.1201-03

**Published:** 2012-05-03

**Authors:** Litha Thomas, Vidya Viswanad

**Affiliations:** Amrita School of Pharmacy, Amrita Vishwa Vidyapeetham University, Kochi, Kerala-682041, India.

**Keywords:** Clotrimazole, Factorial design, Surfactant, Cholesterol, Flux, Entrapment efficiency

## Abstract

The main aim of the study was to develop and statistically optimize the proniosomal gel for enhanced transdermal delivery using 3^2^ factorial designs to investigate the influence of both non-ionic surfactant and cholesterol to maximize the entrapment efficiency and flux. The concentration of non-ionic surfactant and cholesterol were taken as independent variables, while entrapment efficiency and flux were taken as dependent variables. The study showed that the entrapment efficiency depends on both cholesterol and surfactant, whereas permeation flux depends only on the surfactant. Proniosomal gel showed a significantly enhanced drug permeation through the skin, with an enhancement ratio 3.81±1.85 when compared to the drug solution. Comparative evaluation of permeation studies and the *in vitro* release study of optimized proniosomal gel (F5) with that of marketed gel and carbopol gel showed that the penetration of the optimized formulation was enhanced 1.75 times in comparison with that of the marketed formulation, and the release was in a controlled manner. Similarly, the anticandidial activity showed a significantly higher activity (p<0.05) than the marketed and carbopol gel. This may be due to the enhanced penetration of noisome-containing drug through the fungal cell wall, inhibiting the ergo sterol synthesis, thereby causing the fungal cell death due to the presence of penetration enhancer. The stability study at two different temperatures (30 ± 2°C and 4 ± 2°C) confirmed that the formulations were stable even at the end of 45 days. Hence, proniosomal gel is an efficient carrier for the delivery of clotrimazole, thereby prolonging the action.

## Introduction

The main obstacle for the delivery of drug through the skin is the "dead, impermeable barrier devoid of biological activity“ i.e., the stratum cornea; various approaches were put forward for overcoming it [1]. Of these, colloidal carrier is an efficient one as it acts as drug containing reservoirs and can loosen the stratum corneum, thereby modifying the barrier, and can adjust the release rate at the target site [2]. Among the various colloidal carriers, liposome and niosome were the popular ones as they can efficiently encapsulate both hydrophilic and hydrophobic drugs. However, these carriers encountered stability problems, and this led to the discovery of proniosomes. Proniosomes are a liquid crystalline compact niosomal hybrid, which upon hydration will be converted to niosomes and thereby considered the versatile carrier for transdermal delivery [3]. It also provides additional convenience of storage, transport and dosing. The key ingredients used in its preparation are Generally Regarded as Safe [4].

Clotrimazole imidazole antifungal drug has encountered problems such as poor water solubility (0.49 μg/ml), bioavailability, along with the short half-life (2 h), and the drug is lipophilic in nature [5]. So, to overcome these limitations an attempt has been made to incorporate in proniosome and to optimize with respect to entrapment efficiency and transdermal flux for enhanced delivery through the skin upon the variation of surfactant and cholesterol.

## Materials and Methods

The chemicals used were: Clotrimazole was the generous gift from Chettana Pharmaceuticals, Kerala. Cholesterol Extra Pure (SD Fine chemical Ltd, Mumbai), Soya lecithin (Hi-media, Mumbai), Span 60 (Lobachemie Pvt. Ltd, Mumbai), Dialysis Bag (M.W. cut off 12,000–14,000) was purchased from Sigma Aldrich, Bangalore. Sabouraud fluid media and Sabouraud’s Dextrose Agar media from Himedia, Mumbai and DMSO was procured from E.Merck Ltd., Mumbai, India. All other chemicals used were of analytical grade. Candid Gel was purchased from local market.

### Formulation of Clotrimazole-loaded Proniosomal Gel Using Coacervation Technique

Proniosomes were prepared by the coacervation method. The drug with surfactant, lecithin and cholesterol were put in a wide mouth container (the composition is listed in Table 1). To this absolute alcohol was added and container was covered with a lid to prevent loss of solvent from it. The above mixture was warmed on a water bath at 60–70°C until the surfactant mixture dissolved completely. Then to this phosphate buffer of pH 7.4 was added, and the mixture was further warmed in a water bath for 2 min. The above mixture was allowed to cool down at room temperature until the dispersion was converted to proniosomal gel. The final ratio of surfactant: alcohol: aqueous phase was 5:5:4 w/w/w. The prepared gel was preserved in glass container in dark for characterization [6, 7].

### Characterization of Proniosomal Gel

#### Entrapment Efficiency

The clotrimazole-loaded proniosomal gel was hydrated with phosphate buffer and was sonicated in a Sonicator (Equitron, India). The formed clotrimazole-loaded niosomes were separated from unentrapped drug by centrifuging at 17,000 rpm at 4°C for 45 min (Kemi, India). The supernatant was taken and diluted with phosphate buffer. The clotrimazole concentration in the resulting solution was assayed spectrophotometricaly at 261.6nm using UV-1700 (Shimadzu, Japan). The percentage of drug encapsulated was calculated by the following equation [8, 9]:

$$\%\text{EE}=\frac{{\text{C}}_{\text{t}}-{\text{C}}_{\text{f}}}{{\text{C}}_{\text{t}}}\times 100$$

Where, C_t_ = total concentration of drug, C_f_ = concentration of free drug.

#### Ex-vivo Permeation Studies

The histological and biochemical properties of porcine skin have been repeatedly shown to be quite similar to human skin. Furthermore, the stratum corneum, epidermal thickness of pig ear skin are comparable to human skin and also showed similar permeability to human skin[10,11].

#### Permeation Studies

An amount of 100 mg of the proniosomal gel was placed on the donor compartment. The temperature of the receptor vehicle (methanol: PBS of pH 7:4, 20:80 ratio) was maintained at 37 ± 1°C and was constantly stirred by magnetic stirrer. Samples of 500μl were withdrawn from the receptor compartment via the sampling port at different time intervals (0, 1, 2, 3, 4, 5, 6, 7, 8 and 24 h) and analyzed for drug released by UV-1700 (Shimadzu, Japan). The receptor phase was immediately replaced with an equal volume of fresh diffusion buffer. Similarly, the permeation studies for all the formulations and for the drug solution used the control [12–14]. From the permeation, flux of the formulation was compared with that of the control to find out the enhancement ratio.

$$\text{Enhancement Ratio}=\frac{{\text{J}}_{\text{ssenh}}}{{\text{J}}_{\text{sscon}}}$$

#### Experimental Design [15, 16]

A 3^2^ randomized full factorial design was performed, and the amount of surfactant (A) and cholesterol (B) were taken as independent variables, while entrapment efficiency (Y1) and drug permeated (Y2) were taken as dependent variables. The factors were studied at 3 levels (−1, 0, +1) indicating low, medium and high, respectively (Table 2). The statistical optimization procedure was performed with the help of optimization software such as Design Expert 8 (Stat-Ease Inc., Minneapolis, MN, USA) and Statgraphics Centurion 16 (Stat Point Technologies, Inc. Warrenton, Virginia, USA). The software performs response surface methodology (RSM) which includes the multiple regression analysis (MRA), ANOVA and statistical optimization.

#### Multiple Regression Analysis [15]

The use of regression analysis in 3^2^ factorial designs generates different polynomial equations for different models, with interacting terms and regression coefficients, useful in evaluating the responses. The software generates 2 models, particularly full model (non-significant terms included) and reduced model (excluding non-significant terms). In the full model study, the responses were analyzed using the quadratic equation below:

$$\text{Y}={\text{b}}_{0}+{\text{b}}_{1}\text{A}+{\text{b}}_{2}\text{B}+{\text{b}}_{12}\text{AB}+{\text{b}}_{11}{\text{A}}^{2}+{\text{b}}_{22}{\text{B}}^{2}$$

Where Y is the response evaluated, b_0_ is the arithmetic mean response of 9 runs, b_i_ is the estimated coefficient of independent variables.

The main effects (A and B) represent the average result of changing 1 factor at a time from its low to high value. The interaction term (AB) show how the response changes when 2 factors are simultaneously changed. The polynomial terms (A^2^and B^2^) were included to investigate non-linearity.

In the reduced model study, the non-significant terms in the quadratic equation are removed using backward regression procedure to generate a reduced model, which is more important in studying the influence of factors on responses evaluated.

#### ANOVA Study [15]

The software performs the individual analysis of responses and calculates the sum of squares (SS), mean square (MS), Fischer’s ratio (F statistics) and P value. The F statistics and P value give the significance level of each term considering null hypothesis (H_0_) is true. P value less than 0.05 is considered significant at a level of significance α = 0.05.

When the F value obtained is greater than the critical F value from the F distribution table, the factor becomes significant and the null hypothesis is rejected.

#### Vesicular Size Evaluation

The vesicular size of the formulation optimized by statistical optimization was evaluated by Dynamic light Scattering and Scanning Electron Microscopy.

#### Size and Size distribution

Vesicular size distribution studies were evaluated by Dynamic Light Scattering method (Nicomp 380 DLS). 100mg of proniosomal gel was hydrated with 10 ml distilled water with manual shaking. The instrumental setting was fixed as temperature-20°C, viscosity −0.01 poise, and refractive index-1.333[17].

#### Scanning Electron Microscopy

The vesicular size niosomes formed from the hydration of optimized proniosomal gel were evaluated using scanning electron microscopy (Joel jsm-6490la analytical SE). Niosomes formed were mounted on an aluminum stub with double-sided adhesive carbon tape. The vesicles were then sputter-coated with gold using a vacuum evaporator and examined with the scanning electron microscope [4].

#### Zeta Potential determination

Zetasizer (Nicomp 380 ZLS) determined the zeta potential of the optimized formulation. The optimized formulation was hydrated with distilled water and was converted to niosomes; the so formed niosomes were used to determine the zeta potential.

### Comparative Study of Ex-vivo permeation of Optimized Proniosomal Gel with Marketed Gel

Ex-vivo permeation studies of the optimized formulation were compared with the marketed and carbopol gel for 24 h. The permeation flux obtained from the plot of cumulative drug permeated per cm^2^ was plotted against time was used to calculate the enhancement ratio [13, 14].

$$\text{Enhancement Ratio}=\frac{{\text{J}}_{\text{ssenh}}}{{\text{J}}_{\text{sscon}}}$$

### Comparative Study of in vitro Release of Optimized Proniosomal Gel with Marketed and Carbopol Gel

*In vitro* release studies of the optimized formulation were compared with the marketed and carbopol gel for 24 h. The percentage cumulative amount of drug released was noted down and used for the comparison studies.

### In vitro Anticandidal Activity

Microbial growth inhibitory properties of test substances were determined by cup plate method [18]. The formulations (marketed gel, carbopol gel and proniosomal gel) were initially dissolved in DMSO and tested at concentration of 1000 and 500 μg/ml against *Candida albicans* (ATCC 10231).

Sterile Sabouraud’s Dextrose Agar plates were prepared and 0.1 ml of inoculums from standardized culture of test organism was spread uniformly. Wells were prepared by using a sterile borer of diameter 10 mm and 100μl of the test substance and the solvent control were added in each well separately. The plated were placed at 4°C for 1 h to allow the diffusion of test solution into the medium and plated were incubated at 28°C for 48 h, a period of time sufficient for the growth of at least 10 to 15 generation. The zone of inhibition of microbial growth around the well was measured in mm.

### Stability studies [19, 20]

The optimized formulation was kept for stability studies for 45 days at room temperature (30 ± 2°C) and at refrigerator temperature (4 ± 2°C) to determine physical and chemical stabilities. The amount of drug degraded at different time intervals was analyzed. The formulation was evaluated visually and for entrapment efficiency after 7, 15, 30 and 45 days.

## Results and Discussion

The non-ionic surfactant selected for the study is span 60 because of high transition temperature (53°C) and the chemical structure(long alkyl chain), which is reported for the higher entrapment efficiency. Cholesterol is another common additive that acts as cementing agent to improve the stability and play a key role in the entrapment efficiency. Hence, the entrapment efficiency and permeation are largely dependent upon the concentration of non-ionic surfactant and cholesterol, and any alteration in their concentration may lead to the leakiness of the vesicles that result in the leakage of free drug before drug diffusion and fusion of vesicles. Keeping this in mind, the present study focused on investigating the effect of variation in concentration of cholesterol and Span 60 in the entrapment efficiency and permeation. Therefore, the concentration of cholesterol was varied from 15mg to 45 mg and the concentration of Span 60 was varied from 90 mg to 270 mg. Based on this nine formulations were formulated and evaluated for various parameters.

### Optimization of Proniosomal Gel

The dependent variables such as entrapment efficiency and permeation flux calculated are recorded in Table 3.

#### Entrapment Efficiency

Entrapment efficiency is the measure of solute retention. High entrapment efficiency means that less time and effort are needed to remove the unentrapped drug[19]. Vesicular entrapment efficiency is an important parameter that conveys the stability of vesicles and this depends upon the amount of surfactant and amount of cholesterol used. The entrapment efficiency of these formulations varies from 52.22 to 95.40% and was found statistically significant at p<0.05. The entrapment efficiency of various formulations is tabulated in Table 3. From the data in Table 3, it is clear that entrapment efficiency depends upon both surfactant and cholesterol. Effect of independent variable on the entrapment efficiency is represented in the 3D surface plot.

#### Effect of Surfactant amount

Surfactant is an important component in the formation of niosomal vesicles and the variation in the concentration may affect the entrapment efficiency. In the present study, when the concentration of Span 60 was varied from 90mg to 270 mg, the maximum and minimum entrapment efficiency were found to be 95.40% and 52.22%, respectively (from Table 3). The data shows that the variation in the concentration of surfactant from 90mg to 180mg showed a significant increase in the entrapment efficiency (p<0.05), whereas the further increase in concentration from 180 mg to 270 mg decreased the entrapment efficiency.

Initial increase in the concentration of surfactant may increase the number of niosomes formed; therefore, the volume of hydrophobic domain increases and hence increases in entrapment efficiency. However, the further increase in concentration showed decrease in entrapment efficiency, possibly due to formation of mixed micelles along with the niosomal vesicles with high concentration of surfactants, which may lead to lower entrapment efficiency. It is reported that size of micelles < 10nm, thus fewer amounts may be entrapped inside the vesicles [20]. It may be that due to these reasons, it forms vesicles with low entrapment efficiency.

#### Effect of Cholesterol

The concentration of cholesterol plays an important role in the entrapment of drug in the vesicles. The variation in the concentration of cholesterol significantly affects the entrapment efficiency (p<0.05). The observed entrapment efficiency was increased significantly when cholesterol amount was increased from 15mg to 30mg, but further increase in the cholesterol decreased the entrapment efficiency.

The increase in entrapment efficiency shows that the cholesterol, which acts as the “vesicular cement” in the molecular cavities of surfactant bilayer, and abolishes the gel to sol transition, thereby forms less leak vesicles [8]. Therefore, the increase in the rigidity decreases the permeability of the entrapped drug and hence improves the entrapment efficiency. However, when cholesterol amount was increased further from 30mg to 45mg, the opposite result occurred. The reason behind decreased entrapment efficiency may be due to the reason that a cholesterol molecule will compete with drug for the space within the bilayer, remove the drug from the bilayer and in addition to this will disrupt the vesicular membrane structure [8].

#### Ex vivo Permeation Studies

The ex-vivo permeation study gives the information about the behavior of the molecule in-vivo. The amount of the drug permeated gives the information about the amount of drug absorbed into the blood [9]. It is reported that proniosomes will be hydrated to niosomes vesicles before the penetration through the skin. The surfactant and cholesterol amount showed a significant variation in the permeation of drug through the skin.

Steady state fluxes from the proniosomal gel at 24 hours increased with increase in the concentration of surfactant, but cholesterol did not show much significant effect in the permeation. The ex-vivo permeation profile showed a maximum flux value of 10.770±1.245 μg/cm^2^/h and minimum value of 4.319 ±1.059 μg/cm^2^/h. The effect of independent variable on the flux is represented by the 3D surface plot. The increased permeation flux due to increase in surfactant concentration may be due to the non-ionic surfactant present in it, which modifies the structural composition of stratum corneum and increases the thermodynamic activity of the drug as well as skin vesicular partitioning. The enhancement ratio of the formulated proniosomal gel was compared with the drug solution and is represented in Table 4. From the table, it is clear that permeation of the drug loaded in the proniosomal gel is enhanced 1.5–3.7 times in comparison to the drug solution. This may be due to the presence of the non-ionic surfactant, which may alter the strateum corneum and thereby result in enhanced permeation.

#### Best-Fit Model

The optimum formulation of clotrimazole-loaded proniosomal gel was selected on the criteria of attaining maximum transdermal flux and entrapment efficiency. Fitting the data observed in various models, backward fit model was used to predict the best-fit model. The equations were obtained for both coded and actual values of factors. The final equation of reduced model contains only the significant factor terms corresponding to the response analyzed.

Reduced model equation for responses in terms of actual factors:

1. Entrapment Efficiency= 95.10832 + 9.72300 * Surfactant − 6.52553* Cholesterol − 2.05 * Surfactant * Cholesterol − 19.60853 * Surfactant^2^ − 10.33753 * Cholesterol^2^
2. Permeation flux = 7.27671 + 1.86993 * Surfactant.

Reduced model equation for responses in terms of coded factors:

1. Entrapment efficiency = 95.11 + 9.72300 * A − 6.52553 * B − 2.05 * A * B − 19.60853 * A^2^ − 10.33753 * B^2^.
2. Flux = 7.27671 + 1.86993 * A

The inferences from reduced model analysis are: The reduced model also suggests that in case of entrapment efficiency, the increase in surfactant concentration causes the increase in response but the further increase in concentration from the medium to high decreases the entrapment efficiency. In case of the permeation studies, reduced model suggests that the increase in concentration increases permeation but cholesterol does not have any effect on permeation.

#### ANOVA Analysis Report

The ANOVA analysis of the entrapment efficiency is represented in Table 5.

The ANOVA of entrapment efficiency indicates that both surfactant and cholesterol are significant terms as the F values are above the critical F values and thus make the P values less than 0.05 (threshold level). Thus, the null hypothesis H_0_ is rejected and the alternate hypothesis is accepted: both the excipients used significantly influence the entrapment efficiency of clotrimazole-loaded proniosomal gel.

The ANOVA analysis of the permeation flux is represented in Table 6.

The ANOVA of permeation flux indicates that surfactant is significant, as the F value is above the critical F values and thus the P values are less than 0.05 (threshold level). Thus, the null hypothesis H_0_ is rejected and the alternate hypothesis is accepted: the surfactant used significantly influences the permeation flux of clotrimazole-loaded proniosomal gel. 3D response surface plots give a representation of the variations in each response when the two factors are simultaneously changed from lower to higher level. It gives a three dimensional curvature of the change in response at different factor levels. It also gives the variation in design points from the predicted response value.

The formulation with the desirability value closer to unity is chosen as the optimized formulation. The predicted and observed desirability is shown in Table 7.

From the above table it is clear that the desirability value of F5 is closer to unity. Hence, the formulation F5 is considered the optimized formulation.

#### Vesicular Size Evaluation

The vesicular sizes of optimized formulation were evaluated by Dynamic light Scattering and Scanning Electron microscopy.

#### Size and Size distribution

The vesicular size and size distribution were evaluated by using dynamic light scattering. The results showed that vesicular size of the optimized formulation was found to be 316.56±46.79nm.

#### Scanning Electron Microscopy

The vesicular size of the optimized formulation was confirmed to be 250–270nm by scanning electron microscopy.

#### Zeta Potential determination

The magnitude of zeta potential gives a potential stability of the colloidal dispersion. That the particles have large positive or negative charge reveals that they repeal each other and there is dispersion stability. The zeta potential of the optimized formulation showed that the sample is highly stable. It was found as −44.45, and hence this indicates that the prepared formulation is stable.

### Comparison of Ex-vivo permeation study of Optimized Proniosomal Gel with Marketed Gel

The ex-vivo permeation studies clearly indicate that proniosomal gel rate of permeation is enhanced 1.755 times compared to that of the marketed formulation. The increased enhancement ratio in case of proniosomal gel is due to the non-ionic surfactant present in it, which will modify the structural composition of stratum corneum and increase the thermodynamic activity of the drug as well as skin vesicular partitioning.

### Comparative Study of in vitro Release of Optimized Proniosomal Gel with Marketed and Carbopol Gel

The *in vitro* release data after 24 h of optimized formulation was compared with marketed gel and carbopol gel. The release data of all three formulations are tabulated in the table and represented graphically. The cumulative percentage amount of drug release was found to be 70.614± 0.456, 86.959 ± 1.785 and 55.58±1.087%, respectively, for marketed gel, optimized proniosomal gel and carbopol gel at the end of 24 h. In the case of proniosomal gel, more than 85% of drug was released and follows zero order release kinetics. The data shows that proniosomal gel showed that the drug was released in a controlled manner up to 24 h. The proniosomes will be converted to niosomes by the absorption of water and at temperature regulated at 37°C, the drug release should occur from the formed niosomes, hence the release is controlled in the case of proniosomal gel. Marketed clotrimazole gel showed a similar release pattern as that of proniosomal gel until 3^rd^ h. After the initial phase, it produced higher release until 6^th^ h and the released pattern was constant up to 24^th^ h, and cumulative percentage release after 24^th^ h was found to be 70.614± 0.456, whereas in the case of clotrimazole-loaded carbopol gel the release was initially very slow compared to marketed and proniosomal gel and release became constant after 6 h. The cumulative percentage amount of drug released at the end of 24 h was 55.58±1.087%. The statistical analysis by one-way ANOVA showed that the difference in the release pattern of proniosomal, marketed and carbopol gel is statistically significant.

### In vitro Anticandidial Activity

The antifungal activity of optimized formulation against *Candida albicans* was compared with marketed and carbopol gel. The antifungal activity formualtions were carried out at two different concentrations (1000μg and 500μg). The zone of inhibition of various formulations are tabulated in the table and represented graphically.

The antifungal activity of the proniosomal gel was significantly higher than the carbopol and the marketed formulation (p<0.05). This enhanced antifungal activity is due to enhanced penetration of niosome containing drug through the fungal cell wall and inhibiting the ergo sterol synthesis [1].

### Stability Stuides

The stability studies of the optimized formulation at room temperature (30 ± 2°C) and at refrigerator temperature (4 ± 2°C) were carried out for 45 days. The physical appearance showed that it does not show any changes in terms of the freshly prepared formulation. The entrapment efficiency evaluated on 7^th^, 15^th^, 30^th^, 45^th^ day is represented in the table and shows that there are no significant changes in the entrapment during the storage for 45 days in both conditions. However, the formulation is more stable at low temperature compared to room temperature.

## Conclusion

The present study showed that proniosomal gel is a suitable carrier for the delivery of antifungal drug (clotrimazole) with enhanced transdermal delivery. The optimization studies clearly indicated that entrapment efficiency depends both on the concentration of surfactant and cholesterol, and permeation flux depends on the concentration of surfactant only. Hence, the study shows that the formulation enhances the penetration of drug through the skin, and the optimized formulation did not show any formulation problems associated with it. The gel also passes the short term stability studies, indicating the physical and chemical stability of the product.

## Figures and Tables

**Fig. 1:**
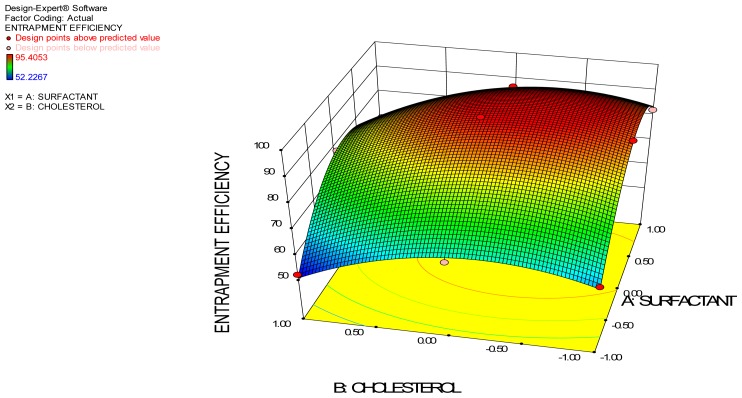
3D Surface Plot for Entrapment Efficiency

**Fig. 2:**
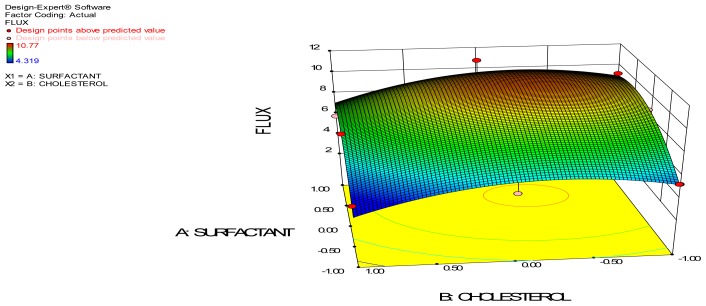
3D Surface Plot of Permeation Flux

**Fig. 3:**
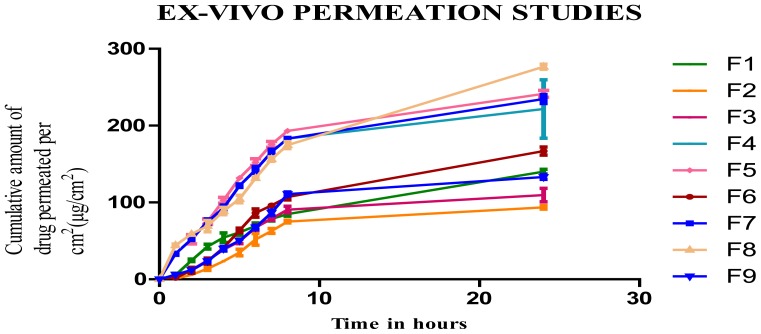
Ex-vivo Permeation Studies of Formulation F1–F9

**Fig. 4:**
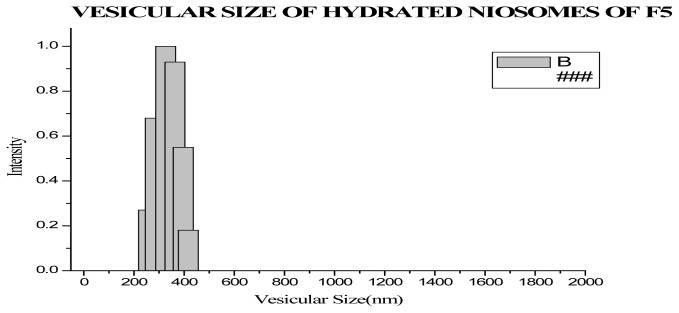
Vesicular Size of Optimized Formulation obtained by DLS

**Fig. 5:**
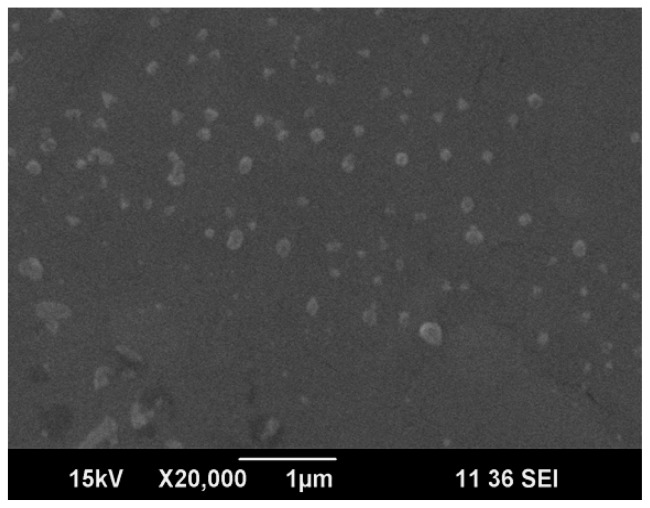
Vesicular Size of Optimized Formulation obtained by SEM

**Fig. 6:**
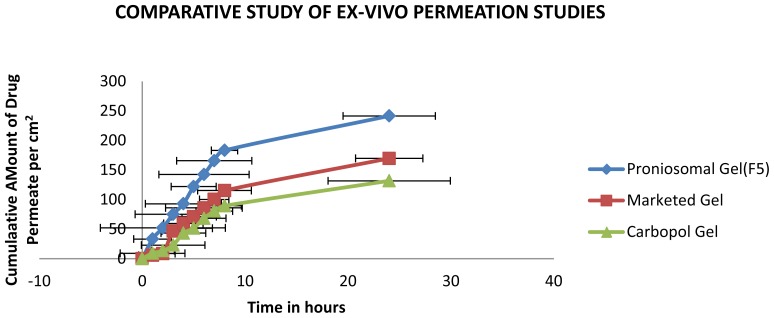
Comparative study of Ex-vivo Permeation studies

**Fig. 7:**
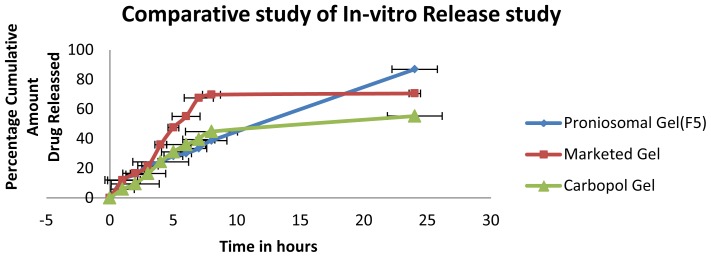
Comparative study of *in vitro* Release study

**Fig. 8:**
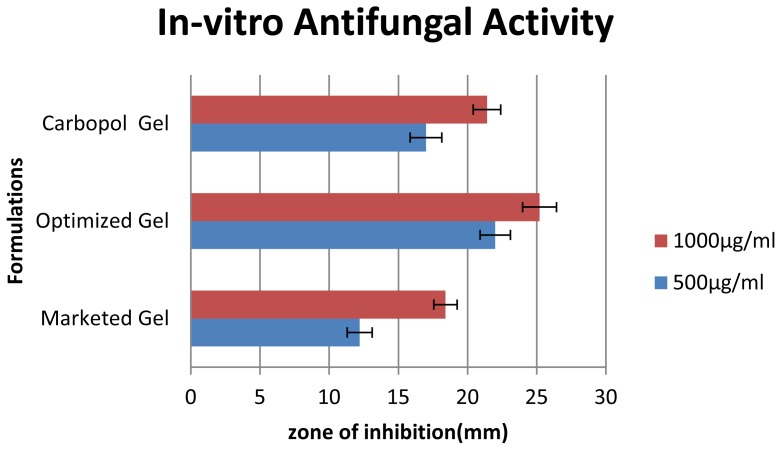
Representing zone of inhibition of *in vitro* Antifungal activity

**Fig. 9:**
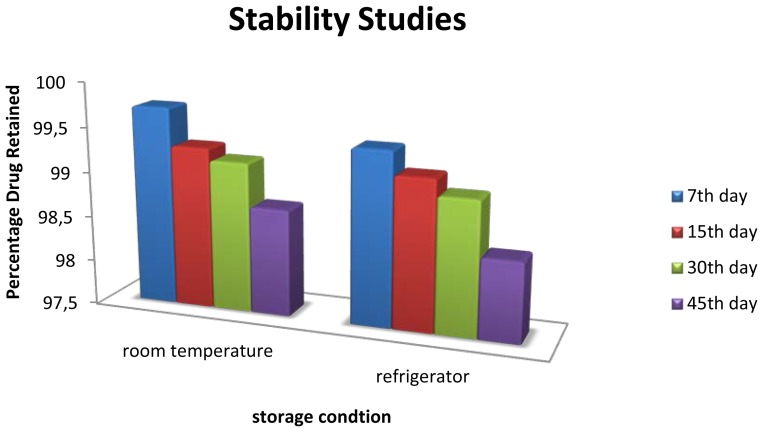
Stability Study of Optimized formulation

**Tab. 1:** Representing the Composition of Formulation

INGREDIENTS	FORMULATION CODE								

	F1	F2	F3	F4	F5	F6	F7	F8	F9
Surfactant (mg)	90	90	90	180	180	180	270	270	270
Cholesterol (mg)	15	30	45	15	30	45	15	30	45
Lecithin (mg)	90	90	90	90	90	90	90	90	90
Ethanol (ml)	0.135	0.3	0.3	0.3	0.3	0.3	0.3	0.3	0.3
Water (ml)	0.1	0.1	0.1	0.1	0.1	0.1	0.1	0.1	0.1
Drug (%)	1	1	1	1	1	1	1	1	1

**Tab. 2:** Correlation of actual and coded factors

FACTOR	CODED	ACTUAL VALUE (mg)	
		SURFACTANT	CHOLESTEROL
LOW	−1	90	15
MEDIUM	0	180	30
HIGH	+1	270	45

**Tab. 3:** Observed Response in 3^2^ Factorial Design for Clotrimazole-loaded Proniosomal Gel

FORMULATION CODE	VARIABLES			

	INDEPENDENT		DEPENDENT	
	
	A	B	Y_1_	Y_2_
F1	−1	−1	60.6023 ± 0.673865	5.244 ± 1.310
F2	−1	0	63.8263 ± 2.79596	4.9254 ± 0.95
F3	−1	1	52.2267 ± 1.555	4.319 ± 1.059
F4	0	−1	91.7233 ± 1.54043	8.60 ± 2.087
F5#	0	0	95.4053 ± 1.40069	9.453 ± 2.022
F6	0	1	77.5213 ± 2.00448	7.241 ± 0.317
F7	1	−1	82.3463 ± 1.55203	9.163 ± 1.045
F8	1	0	86.8763 ± 0.553715	10.770 ± 1.245
F9	1	1	65.7707 ± 2.26477	5.775 ± 0.438

* The values are expressed as Mean ± SD; n = 3,

# Optimized Formulation.

**Tab. 4:** Representing the Flux and Enhancement Ratio of the Proniosomal Gel

FORMULATION CODE	PERMEABILITY FLUX	ENNHANCEMENT RATIO
F1	5.244 ± 1.310	1.76
F2	4.9254 ± 0.95	1.65
F3	4.319 ± 1.059	1.45
F4	8.60 ± 2.087	2.89
F5	9.453 ± 2.022	3.27
F6	7.241 ± 0.317	2.43
F7	9.163 ± 1.045	3.07
F8	10.770 ± 1.245	3.61
F9	5.775 ± 0.438	1.93
Drug Solution	2.98±0.52	

**Tab. 5:** ANOVA Analysis of Entrapment Efficiency

Source	Sum of Squares	Df	Mean Square	F-Ratio	P-Value
A:Surfactant	567.22	1	567.22	155.64	0.0011*
B:Cholesterol	255.496	1	255.496	70.10	0.0036*
AA	768.989	1	768.989	211.00	0.0007*
AB	16.81	1	16.81	4.61	0.1210
BB	213.729	1	213.729	58.64	0.0046*
Total error	10.9335	3	3.64451		
Total (corr.)	1833.18	8			

* Significant values.

**Tab. 6:** ANOVA Analysis of Permeation Flux

Source	Sum of Squares	Df	Mean Square	F-Ratio	P-Value
A:Surfactant	20.9799	1	20.9799	17.73	0.0245*
B:Cholesterol	5.36193	1	5.36193	4.53	0.1232
AA	5.99919	1	5.99919	5.07	0.1098
AB	1.51659	1	1.51659	1.28	0.3399
BB	5.50545	1	5.50545	4.65	0.1199
Total error	3.55046	3	1.18349		
Total (corr.)	42.9135	8			

* Significant values.

**Tab. 7:** Desirability correlation of formulations

FORMULATION CODE	PREDICTED DESIRABILITY	OBSERVED DESIRABLITY
F1	0.00720162	0.00436896
F2	0.0135459	0.027096
F3	0.0000465304	0.0
F4	0.632078	0.626386
F5	0.865704*	0.855237*
F6	0.165684	0.170966
F7	0.320917	0.365422
F8	0.524163	0.426302
F9	0.0260801	0.0329907

* significant formulation.

**Tab. 8:** Evaluation *in vitro* Antifungal Activity by Zone of Inhibition

		Zone of inhibition of Test samples(mm)		
		
Microorganism	Concentr. (μg/ml)	Marketed (K1)	Optimized Formulation (K3)	Carbopol gel (K4)
*Candida albicans*	1000	18.4 ± 0.89	25.2 ± 1.10	21.4 ± 1.14
	500	12.2 ± 0.84	22 ± 1.22	17 ± 1

The values are expressed as Mean ± SD; n = 5.

**Tab. 9:** Stability Study of Optimized formulation

TEMPERATURE	PERCENTAGE DRUG RETAINED (%)			
	
	*7^th^* *Day*	*15^th^* *Day*	*30^th^* *Day*	*45^th^* *Day*
Refrigerator (4 ± 2°C)	99.72	99.30	99.17	98.69
Room Temperature (30 ± 2°C)	99.43	99.17	98.99	98.39
